# Research on the Influence of the Interfacial Properties Between a Cu_3_BiS_3_ Film and an In*_x_* Cd_1−_ *_x_* S Buffer Layer for Photoelectrochemical Water Splitting

**DOI:** 10.1002/advs.202204029

**Published:** 2022-10-17

**Authors:** Xiaomin Wu, Weidong Zhao, Yucheng Hu, Guohong Xiao, Huanyang Ni, Shigeru Ikeda, Yunhau Ng, Feng Jiang

**Affiliations:** ^1^ Institute of Hydrogen Energy for Carbon Peaking and Carbon Neutralization School of Semiconductor Science and Technology South China Normal University Foshan 528225 China; ^2^ Institute of Semiconductor Science and Technology South China Normal University 55 Zhongshan Avenue West, Tianhe District Guangzhou 510631 China; ^3^ Key Laboratory of Polar Materials and Devices Ministry of Education East China Normal University Information Building, 500 Dongchuan Road Shanghai 200241 China; ^4^ Department of Chemistry Konan University 9‐1 Okamoto, Higashinada Kobe Hyogo 658–8501 Japan; ^5^ School of Energy and Environment City University of Hong Kong Kowloon Hong Kong 999077 China

**Keywords:** band alignment, Cu_3_BiS_3_ compound semiconductors, photovoltaic thin film, solar water splitting

## Abstract

The ternary compound photovoltaic semiconductor Cu_3_BiS_3_ thin film‐based photoelectrode demonstrates a quite promising potential for photoelectrochemical hydrogen evolution. The presented high onset potential of 0.9 *V*_RHE_ attracts much attention and shows that the Cu_3_BiS_3_ thin films are quite good as an efficient solar water splitting photoelectrode. However, the CdS buffer does not fit the Cu_3_BiS_3_ thin film: the conduction band offset between CdS and Cu_3_BiS_3_ reaches 0.7 eV, and such a high conduction band offset (CBO) significantly increases the interfacial recombination ratio and is the main reason for the relatively low photocurrent of the Cu_3_BiS_3_/CdS photoelectrode. In this study, the In*_x_* Cd_1−_ *_x_* S buffer layer is found to be significantly lowered the CBO of CBS/buffer and that the In incorporation ratio of the buffer influences the CBO value of the CBS/buffer. The Pt‐TiO_2_/In_0.6_Cd_0.4_S/Cu_3_BiS_3_ photocathode exhibits an appreciable photocurrent density of ≈12.20 mA cm^−2^ at 0 *V*_RHE_ with onset potential of more than 0.9 *V*_RHE_, and the ABPE of the Cu_3_BiS_3_‐based photocathode reaches the highest value of 3.13%. By application of the In_0.6_Cd_0.4_S buffer, the Cu_3_BiS_3_‐BiVO_4_ tandem cell presents a stable and excellent unbiased STH of 2.57% for over 100 h.

## Graphical abstract

Cu_3_BiS_3_ presents significant photoelectrochemical properties, but traditional CdS buffer does not fit Cu_3_BiS_3_ due to its high CBO value. Here In*_x_* Cd_1−_ *_x_* S buffer is found to effectively lower the CBO at the interface of Cu_3_BiS_3_/buffer. The Pt‐TiO_2_/In_0.6_Cd_0.4_S/Cu_3_BiS_3_ photocathode exhibits the highest ABPE of 3.13% and the Cu_3_BiS_3_‐BiVO_4_ tandem cell presents unbiased STH of 2.57% for over 100 h.

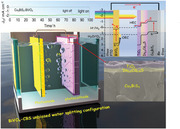

## 1 Introduction

It is very emergent to provide a renewable alternative for the consumption of petroleum to meet the current and future power requirements of the world. Conversion of abundant solar energy to sustainable and storable hydrogen energy is an attractive approach.^[^ ^1^ ^]^ Photoelectrochemical water splitting is a promising way to meet energy and environmental issues.^[^ ^2^ ^]^ Since Honda and Fujishima observed the photocatalysis effect of TiO_2_ in 1972, a lot of materials with various patterns and different structures have been reported for photocatalysis and photoelectrocatalysis.^[^ ^3^ ^]^ Among them, using solar energy to produce hydrogen through photoelectrochemical (PEC) water splitting, which is driven by the electrons and holes in semiconductor photoelectrodes, is one of the most promising ways to yield green and sustainable fuels in the future.^[^ ^4^ ^]^ It should be noted here that the selection or design of materials or structures for solar water splitting photoelectrodes should be in reference to photovoltaic devices. Previously reported highly successful record solar water splitting efficiencies by photovoltaic‐structured photoelectrodes, such as CuIn*_x_* Ga_1−_ *_x_* Se_2_(CIGS), Cu_2_ZnSnS_4_(CZTS), Cu(InGa)Se (CIS), Si and GaAs, have already proven that efficient PEC solar water splitting photoelectrodes should have photovoltaic structures, such as CIGS, CZTS or Si thin film solar cells. Xingwang Zhang et al. reported a record solar‐to‐hydrogen (STH) efficiency using NiCoSe*_x_* based on a Si thin film photoelectrode by reference to a Si thin film solar cell in 2016.^[^ ^5^ ^]^ After that, a record STH efficiency of 13.13% was achieved by a heterojunction of GaAs/Al*_x_* Ga_1−_ *_x_* As, which is very similar to its photovoltaic structure.^[^ ^6^ ^]^ By reference to CIGS thin film solar cell, Prof. Domen and his group reported a CIGS thin film photocathode with a STH efficiency of over 11%.^[^ ^7^ ^]^ In addition, Todd G. Deutsch et al. also reported a record STH efficiency over 16% obtained from a GaAs thin film photovoltaic‐based photocathode.^[^ ^8^ ^]^ Thus, several typical photovoltaic thin film materials have been investigated for application in solar water splitting photoelectrodes. For example, CZTS photovoltaic thin films were found to be an excellent photocathode for solar water splitting by the Domen group in 2010.^[^ ^9^ ^]^ In recent years, our group has significantly developed the potential of CZTS thin film photovoltaics for solar water splitting.^[^ ^10^ ^]^ Jooho Moon and Tilley et al. reported several impressive results using emerging Sb_2_Se_3_ film photovoltaic materials for solar water splitting.^[^ ^11^ ^]^ Recently, we have developed an interesting ternary copper‐based compound: Cu_3_BiS_3_, and this compound has been used as a photovoltaic film for solar water splitting. The Cu_3_BiS_3_ photovoltaic film‐based photocathode presents a remarkable onset potential over 0.9 *V*_RHE_ with excellent photoelectrochemical current densities (≈7 mA cm^−2^ at 0 *V*_RHE_).^[^ ^12^ ^]^ This high onset potential of the Cu_3_BiS_3_ film‐based photocathode has very significant value for photoelectrochemical water splitting devices, especially for the photocathode–photoanode tandem cell for standalone solar water splitting. The Cu_3_BiS_3_ photovoltaic film‐based photoelectrode presented an especially high onset potential. We believe that this Cu_3_BiS_3_ photocathode will attract much attention from various researchers and should be widely studied in the future. However, a lot of existing problems of the Cu_3_BiS_3_ photoelectrodes have not been resolved or studied. One of the biggest issues we found in the Cu_3_BiS_3_ photoelectrode is that the band alignment of Cu_3_BiS_3_ does not fit the CdS buffer layer well. The band cliff offset value between Cu_3_BiS_3_ and CdS is over 0.7 eV, resulting in a serious interfacial recombination of the photoexcited carriers between Cu_3_BiS_3_ and the CdS buffer. In this work, we found that the In*_x_* Cd_1−_ *_x_* S buffer layer significantly lowered the CBO of the CBS/buffer, largely decreasing the interfacial recombination ratio of the Cu_3_BiS_3_ film‐based photocathode for solar water splitting. It was found that the Pt‐TiO_2_/In_0.6_Cd_0.4_S/Cu_3_BiS_3_ photocathode exhibited an appreciable photocurrent density of ≈12.20 mA cm^−2^ at 0 *V*_RHE_ with onset potential of more than 0.9 *V*_RHE_, and the ABPE of our Cu_3_BiS_3_‐based photocathode reached the highest value of 3.13% to date. In addition, this photocathode has a long‐term high stability of over 10 h. By application of the In_0.6_Cd_0.4_S buffer, the Cu_3_BiS_3_‐BiVO_4_ tandem cell presented a stable and excellent unbiased STH of 2.57% for over 100 h, which is the highest STH for Cu_3_BiS_3_ devices. To the best of our knowledge, this is an excellent strategy for elaborately introducing the In*_x_* Cd_1−_ *_x_* S thin layer to modify the Cu_3_BiS_3_ layer to solve the bandgap match problem of the buffer layer and Cu_3_BiS_3_ to achieve outstanding PEC performance.

## 2 Results and Discussion

### 2.1 Surface and Cross Section SEM Images of the Cu_3_BiS_3_‐Based Photocathode

**Figure 1:**
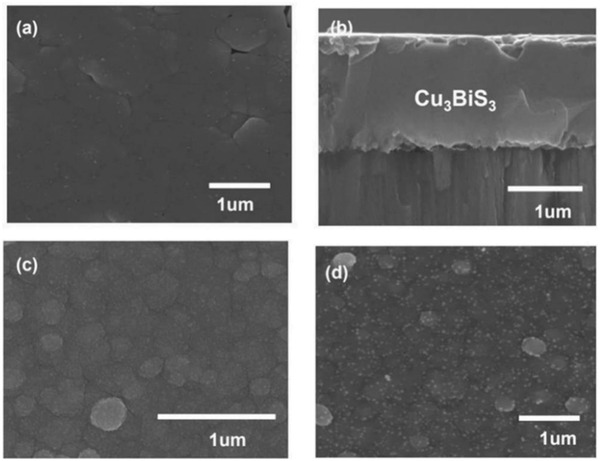
Surface and cross‐section SEM images of the Cu_3_BiS_3_‐based photocathode: a) the surface morphology of Cu_3_BiS_3_; b) cross‐section of Cu_3_BiS_3_; c) the surface morphology of the In_0.6_Cd_0.4_S‐covered Cu_3_BiS_3_; d) the surface morphology of the Pt‐TiO_2_/In_0.6_Cd_0.4_S/Cu_3_BiS_3_.

In this work, the Cu_3_BiS_3_ film was prepared by spray pyrolysis on a Mo‐coated glass substrate.^[^ ^13^ ^]^ **Figure** **1**a,b shows SEM images of the surface and cross‐section of the Cu_3_BiS_3_ film, and we observed that the Cu_3_BiS_3_ film has well‐grown grains that are closely arranged and smooth on the substrate, with a grain size of ≈1 µm. Furthermore, the crystallinity of Cu_3_BiS_3_ was confirmed by XRD pattern analyses, as shown in Figure S1, Supporting Information. We found that the Cu_3_BiS_3_ film was well crystallized but still existed in a very small amount of secondary phases. Then, from the X‐ray photoelectron spectroscopy (XPS) we found the peaks from Cu 2p (Figure S2a, Supporting Information), Bi 4f (Figure S2b, Supporting Information) and S 2p (Figure S2c, Supporting Information) are well according to the previously reported data from Cu_3_BiS_3_ compound^[^ ^14^ ^]^ and the EDS results (Figure S2d, Supporting Information) shown that the element ratio of Cu, Bi and S are 42.87%, 13.94% and 43.19%, respectively. The Cu/Bi/S ratio is well according to the stoichiometric ratio of Cu_3_BiS_3_.

The surface of the Cu_3_BiS_3_ film was modified by chemical bath deposition.^[^ ^15^ ^]^ Figure 1c shows the surface of Cu_3_BiS_3_ modified by In_0.6_Cd_0.4_S. It can be clearly seen from the figure that the Cu_3_BiS_3_ surface with a little gap before was filled by a buffer layer of In_0.6_Cd_0.4_S and formed the morphology of the In_0.6_Cd_0.4_S coating. However, the overall structural morphology of the electrode remained unchanged and was still a relatively flat and compact plane structure. Figure 1d shows that the electrode surface was covered by TiO_2_. It can be seen from the figure that the surface of the sample was smoother than before. It is worth mentioning that a smooth surface between layers is beneficial to the charge transfer between interfaces.^[^ ^16^ ^]^

To study the distribution of elements on the TiO_2_/In_0.6_Cd_0.4_S/Cu_3_BiS_3_ photocathode, EDX elemental analysis was carried out on the cross‐section of TiO_2_/In_0.6_Cd_0.4_S/Cu_3_BiS_3_. As shown in Figure S3, Supporting Information, we found that Cu, Bi, S, In, Cd and Ti were evenly distributed among the layers.

Then, the In*_X_* Cd_1−_ *_X_* S was confirmed by XPS analyses, as shown in Figure S4, Supporting Information. We found that the binding energy of In_0.6_Cd_0.4_S has shifted compared with In_2_S_3_ and CdS, indicating the formation of In‐Cd‐S ternary compound instead of In_2_S_3_‐CdS hetero‐structure.^[^ ^17^ ^]^ Besides, from the Figure S5, Supporting Information, we could see that high‐resolution HRTEM image of Cu_3_BiS_3_ was covered with different buffer layers of CdS (Figure S5a, Supporting Information) and In_0.6_Cd_0.4_S (Figure S5c, Supporting Information). The observed lattice spacings of 0.285, 0.30 and 0.33 nm were corresponding to the respective interlayer distance of (1 3 1) (1 0 2) and (2 0 1) plane of orthorhombic Cu_3_BiS_3_ (PDF 43–1479) which were consistent with XRD. Moreover, the lattice spacings of 0.245 nm was consistent with the standard values of (1 0 2) plane of the hexagonal CdS (PDF41‐1049) and the lattice spacings of 0.276 nm was between 0.270 (In_2_S_3_; PDF33‐0624) and 0.316 nm (CdS; PDF41‐1049). Further, in order to understand the interface between Cu_3_BiS_3_ and buffer layers, we performed a Geometric Phase Analysis (Figure S5b,d). It was found that the interface of Cu_3_BiS_3_/In_0.6_Cd_0.4_S exhibited a small compressive strain compared with Cu_3_BiS_3_/CdS, while the interface of Cu_3_BiS_3_/In_0.6_Cd_0.4_S did not show significant tensile strain along the *xy* direction, indicating that Cu_3_BiS_3_/In_0.6_Cd_0.4_S had a better interaction than Cu_3_BiS_3_/CdS.^[^ ^18^ ^]^

### 2.2 Optical Properties of the Photocathodes

To further analyze the band structure of the prepared Cu_3_BiS_3_ electrode, the optical band gap of Cu_3_BiS_3_ was researched. According to previous reports, Cu_3_BiS_3_ is a direct bandgap.^[^ ^14^ ^]^ As shown in **Figure** **3**a, the optical band gap of Cu_3_BiS_3_ was 1.67 eV, which made Cu_3_BiS_3_ a potential research object for the construction of an efficient PEC–Hydrogen Evolution Reaction (HER) system.

**Figure 2:**
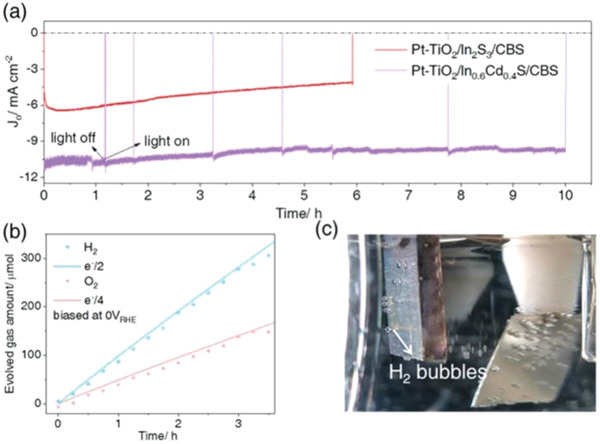
PEC performances of the Cu_3_BiS_3_‐based photocathodes. a) Photocurrent density time curve; b) hydrogen and oxygen evolution amounts of the Pt‐TiO_2_/In_0.6_Cd_0.4_S/Cu_3_BiS_3_ photocathode with various illumination times; c) photograph of the Pt‐TiO_2_/In_0.6_Cd_0.4_S/Cu_3_BiS_3_ photocathode under working conditions. All measurements were carried out in a 0.2 mol L^−1^ Na_2_HPO_4_/NaH_2_PO_4_ solution (pH 6.5) under AM 1.5 G simulated solar light irradiation.

**Figure 3:**
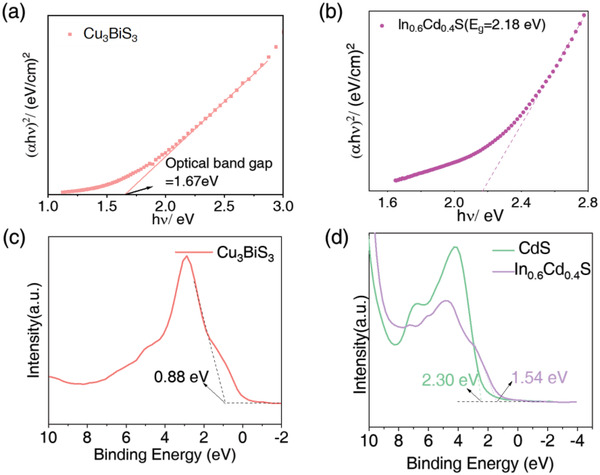
Optical properties of the photocathodes. a) Tauc curve of Cu_3_BiS_3_/glass; b) Tauc curve of In_0.6_Cd_0.4_S/glass; c) VB‐XPS of Cu_3_BiS_3_; d) VB‐XPS of CdS and In_0.6_Cd_0.4_S.

In our work, the buffer layer of the photocathode was the In*_X_* Cd_1−_ *_X_* S film, which not only compensates for the narrow band gap of the In_2_S_3_ thin film shortcoming and increases the transmittance but also has the characteristics of an adjustable band gap which can be used as a buffer layer material for many photocathode heterojunctions.^[^ ^19^ ^]^ First, its optical properties were intensively investigated. As shown in Figure S6, Supporting Information, the absorbance of the In*_x_* Cd_1−_ *_x_* ternary sulfide decreased significantly from 400 to ≈650 nm and finally fluctuated within a narrow range. In addition, we observed that the absorptivity of the In_0.6_Cd_0.4_S film was the lowest relatively, indicating that the In_0.6_Cd_0.4_S film was more suitable as a buffer layer since it will lead to an improved conversion efficiency of the photocathode. Furthermore, according to the Kubelka–Munk equation and combined with previous report^[^ ^20^ ^]^ which learned that CdS, In*_x_* Cd_1−_ *_x_* are direct bandgap semiconductors and In_2_S_3_ is an indirect bandgap semiconductor. We calculated the Tauc diagram as shown in Figure 3b, Figure S7, Supporting Information. We found that the band gaps of CdS, In_0.5_Cd_0.5_S, In_0.6_Cd_0.4_S, In_0.7_Cd_0.3_S and In_2_S_3_ were 2.39, 2.22, 2.18, 2.13 and 2.00 eV, respectively.

Besides, in order to confirm the accuracy of the band structure, we considered using the VB‐XPS method to measure the valence band potential (*E*_VB,XPS_) measured by VB‐XPS plots.^[^ ^14^, ^18^, ^20^ ^]^ The corresponding *E*_VB,XPS_ of Cu_3_BiS_3_, CdS, In_0.5_Cd_0.5_S, In_0.6_Cd_0.4_S, In_0.7_Cd_0.3_S and In_2_S_3_ were measured to be 0.88, 2.30, 1.79, 1.54, 1.60 and 1.70 eV, respectively (Figure 3c,d and Figure S8a–c, Supporting Information). Then, the *E*_VB_ of the corresponding standard hydrogen electrode (*E*_VB,NHE_) can be calculated. It according to the following formula:^[^ ^21^ ^]^ *E*_VB,NHE_ = *φ* + *E*_VB,XPS_ − 4.44, where *φ* is the work function of the instrument (4.20 eV). Thus, the *E*_VB,NHE_ of Cu_3_BiS_3_, CdS, In_0.5_Cd_0.5_S, In_0.6_Cd_0.4_S, In_0.7_Cd_0.3_S and In_2_S_3_ were calculated to be 0.64, 2.06, 1.55, 1.30, 1.36 and 1.46 eV. Then, according to the *E*_CB,NHE_ = *E*_VB,NHE_ − *E*_g_, we calculated the *E*_CB,NHE_ as shown in Table S1, Supporting Information.

Furthermore, Mott–Schottky analysis was performed. The negative slope in the curve in Figure S9, Supporting Information, clearly shows that Cu_3_BiS_3_ is a p‐type semiconductor.^[^ ^22^ ^]^ For p‐type semiconductors, *E*_fb_ is usually near the valence band (VB). That is, the flat band potential is 0.1–0.2 eV lower than the VB potential.^[^ ^19^ ^]^ And our results through the XPS test were within this range. Moreover, to further understand the carrier transfer of the buffer layer in the photocathode, the Mott–Schottky test was used for analysis (as shown in Figure S8d, Supporting Information). First, the positive slope clearly indicates that In*_x_* Cd_1−_ *_x_* S is an n‐type semiconductor^[^ ^23^ ^]^ and shows the flat band (*E*_fb_) positions of all samples. We found that the Fermi levels of CdS, In_0.5_Cd_0.5_S, In_0.6_Cd_0.4_S, In_0.7_Cd_0.3_S and In_2_S_3_ were −0.53, −0.82, −1.10, −0.98 and −0.70 *V*_RHE_, respectively. Notably, the most negative *V*_fb_ of −1.10 *V*_RHE_ was from In_0.6_Cd_0.4_S, which implies that it has the highest energy level of photogenerated electrons and further demonstrated that it can enhance the built‐in electric field in the space charge region, promote the separation of photogenerated electron hole pairs and make the carriers more easily transfer from the photocathode, thus enhancing the HER.^[^ ^24^ ^]^

Finally, we can learn the carrier density from the following formula:

(1) $$N=\left(\frac{2}{\varepsilon_{0}{\varepsilon e}_{0}}\right)\left[\frac{1}{\frac{d\left(\frac{1}{C^{2}}\right)}{dV}}\right]$$

where *N* is the interface charge density, e_0_ is the electron charge, *ε*_0_ is the vacuum dielectric constant, *ε* is the relative dielectric constant, and d(1/*C* ^2^)/d*V* is the slope of the Mott–Schottky curve. Figure S8d and **Table** **1** shows that In_0.6_Cd_0.4_S has the lowest slope, which indicates that its carrier concentration is relatively high and suggests that the buffer layer of In_0.6_Cd_0.4_S has excellent electrical performance and will improve the ability of electrons to be extracted from the Cu_3_BiS_3_ absorption layer and then facilitate the proton reduction reaction.^[^ ^25^ ^]^

**Table 1:** PEC performance of previously reported typical photocathodes and their tandem cell modules of photocathodes‐BiVO_4_

Typical photocathodes	*J* [mA cm^−2]^; ABPE; stability; electrolyte pH	Onset potential/*V*_RHE_	Tandem cell STH; stability	Reference (Ref.)
Cu_3_BiS_3_/ln_0.6_Cd_0.4_S/TiO_2_‐Pt	−10.80 (0 *V*_RHE_);2.41%; 10 h; (pH 6.5)	0.90	2.57%; 100 h	This work
CuIn_0.5_Ga_0.5_Se_2_/CdS‐Pt	−28.00 (0 *V*_RHE_); 12.50%; NR; (pH 6.8)	0.70	3.70%; 10 min	^[^ ^7^ ^]^; Energy Environ. Sci.
Perovskite/IO‐TiO_2_‐H_2_ase	−5.00 (0 *V*_RHE_); NR; 12 h; (pH 6.0)	0.80	1.10%; 10 h	^[^ ^26^ ^]^; ACS Energy Lett.
Cu_3_BiS_3_/CdS/TiO_2_‐Pt	−7.00 (0 *V*_RHE_); 1.70%; 10 h; (pH 6.5)	0.90	2.04%; 20 h	^[^ ^12^ ^]^; Nat. Commun.
FTO/Au/SnS/CdS/TiO_2_/Pt	−23.00 (0 *V*_RHE_); NR; NR; (pH 9.0)	0.30	1.70%; 24 h	^[^ ^27^ ^]^; Adv. Sci.
Zn:InP NW/TiO_2_‐Pt	−18.00 (0 *V*_RHE_);4.0%; 10 h; (pH 1.0)	0.60	0.50%; 1 min	^[^ ^28^ ^]^; ACS Nano
P‐BVO/Pt	−0.30 (0 *V*_RHE_); NR; 24 h; (pH 7.0)	1.23	0.14%; 1.5 h	^[^ ^29^ ^]^; Adv. Funct. Mater.
Sb_2_Se_3_/CdS/TiO_2_‐Pt	−20.00 (0 *V*_RHE_); 3.40%; NR; (pH 1.0)	0.50	1.50%; 10 h	^[^ ^19^ ^]^; Appl. Catal. B
Cu/Cu_2_O/Ga_2_O_3_/TiO_2_/NiMo	−10 (0 *V*_RHE_);NR; 100 h; (pH 5.0)	1.0	3%; 12 h	^[^ ^30^ ^]^; Nat. Catal.
CuBi_2_O_4_	−3.70 (0.4 *V*_RHE_); NR; NR; (pH 6.8)	1.00	0.86%; 1 h	^[^ ^31^ ^]^; ACS Appl. Mater. Interfaces
Si(n+‐p)‐Mo‐Ni	−35.00 (0 *V*_RHE_); NR; NR; (pH 1.0)	0.50	3.00%; 2 h	^[^ ^32^ ^]^; ACS Sustain. Chem. Eng.

### 2.3 The Band Alignment of the Cu_3_BiS_3_‐Based Photocathode Modified with Different Buffers

**Figure** **5** shows the band alignment of the In*_X_* Cd_1−_ *_X_* S/Cu_3_BiS_3_ heterojunction. The results showed that the band structure of In*_X_* Cd_1−_ *_X_* S/Cu_3_BiS_3_ belongs to the type II heterojunction. This type can effectively reduce electron hole pair coincidence and improve photocatalytic efficiency in the field of photocatalytic water splitting.^[^ ^33^ ^]^ As seen from the figure, CBO tends to decrease with introducing suitable In element into CdS.

**Figure 4:**
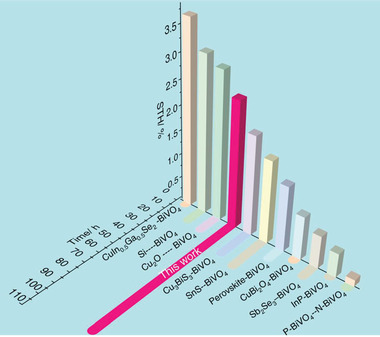
Efficiency and stability benchmarks for previously reported photocathode–photoanode tandem cells (specific parameters are shown in Table 1).

**Figure 5:**
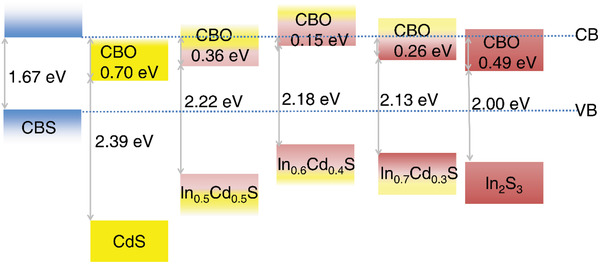
The band alignment of the Cu_3_BiS_3_‐based photocathode modified with different buffers. Details are in Table S1, Supporting Information.

It is worth mentioning that the CBO value of pure CdS/Cu_3_BiS_3_ is too large and will increase the interface barrier and hinder photoelectron transfer.^[^ ^34^ ^]^ Therefore, in this case, the optimal CBO of In_0.6_Cd_0.4_S/Cu_3_BiS_3_ is 0.15 eV, which will accelerate the transfer of electrons and effectively reduce the photoexcited electron–hole pair recombination.^[^ ^34^ ^]^

### 2.4 PEC Performances of the Cu_3_BiS_3_‐Based Photocathodes

The PEC performance of the Pt‐TiO_2_/In*_x_* Cd_1−_ *_x_* S/Cu_3_BiS_3_ photocathode varies with the molar ratio of In:Cd in the buffer layer, as discussed by LSV. In particular, the Cu_3_BiS_3_ acts as the absorption layer, and In*_x_* Cd_1−_ *_X_* S acts as the buffer layer. From the positive slope of Mott–Schottky of Cu_3_BiS_3_ (Figure S9, Supporting Information), Cu_3_BiS_3_ was observed to be a p‐type semiconductor, while from the negative slope of Mott–Schottky of In*_x_* Cd_1−_ *_X_* S (Figure S8d, Supporting Information), the buffer layers were shown to be an n‐type semiconductor. The p‐n heterojunction formed between Cu_3_BiS_3_/In*_x_* Cd_1−_ *_x_* S_,_ and this structure can improve the separation efficiency of photogenerated carriers.^[^ ^35^ ^]^ After all, in this composite photocathode film, TiO_2_ has a photochemical corrosion prevention function, which can play a photocathodic protection role for semiconductors in corrosive media.^[^ ^36^ ^]^ Moreover, Pt catalytic particles are photodeposited on top of TiO_2_ due to the work function of Pt atoms being higher than that of TiO_2_. After the combination of Pt and TiO_2_, the electrons on TiO_2_ are transferred to Pt atoms until their Fermi energy levels are equal, thus promoting the separation of electron–hole pairs and further improving photocatalytic efficiency.^[^ ^37^ ^]^

**Figure 6:**
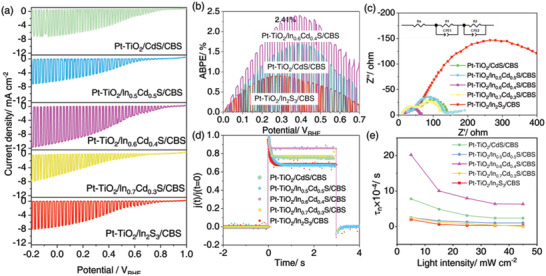
PEC performances of the Cu_3_BiS_3_‐based photocathodes. a) Chopped photocurrent density–potential curves of the Pt‐TiO_2_/CdS/Cu_3_BiS_3_, Pt‐TiO_2_/In_0.5_Cd_0.5_S/Cu_3_BiS_3_, Pt‐TiO_2_/In_0.6_Cd_0.4_S/Cu_3_BiS_3_, Pt‐TiO_2_/In_0.7_Cd_0.3_S/Cu_3_BiS_3_, and Pt‐TiO_2_/In_2_S_3_/Cu_3_BiS_3_; b) ABPE curves; c) a representative Nyquist plot obtained from the EIS measurement; d) the transit photocurrent spectra; e) the lifetime of carriers generated of Pt‐TiO_2_/CdS/Cu_3_BiS_3_, Pt‐TiO_2_/In_0.5_Cd_0.5_S/Cu_3_BiS_3_, Pt‐TiO_2_/In_0.6_Cd_0.4_S/Cu_3_BiS_3_, Pt‐TiO_2_/In_0.7_Cd_0.3_S/Cu_3_BiS_3_, and Pt‐TiO_2_/In_2_S_3_/Cu_3_BiS_3_ from IMVS (details are in Figure S13, Supporting Information). All measurements were carried out in a 0.2 mol L^−1^ Na_2_HPO_4_/NaH_2_PO_4_ solution (pH 6.5) under AM 1.5 G simulated solar light irradiation.

Prior to this work, we researched the influence of the buffer layer thickness on PEC performance. As shown in Figure S10, Supporting Information, we can see that buffer layers of In*_x_* Cd_1−_ *_x_* S showed the best PEC performance when the deposition time was 10 min and the buffer layer of In_2_S_3_ was suitable for 20 min. From our previous work, we learned that the best deposition time of CdS was 15 min.^[^ ^12^ ^]^ After determining the optimal deposition time for each ratio, we proceeded with subsequent work. As we predicted, the Pt‐TiO_2_/In*_x_* Cd_1−_ *_x_* S/Cu_3_BiS_3_ photocathode of the buffer layer was In_0.6_Cd_0.4_S, and this photocathode exhibited the highest photocurrent density and onset potential: a 10.80 mA cm^−2^ photocurrent density at 0 *V*_RHE_, with a 0.9 *V*_RHE_ onset potential value and its conversion efficiency (ABPE) is 2.41%, which is the highest world record of Cu_3_BiS_3_‐based photocathodes (as shown in **Figure** **6**a,b). Furthermore, to evaluate the repeatability and scientific of our work, several replications were conducted, as shown in Figure S11, Supporting Information. Comparatively, samples with more In showed higher PEC performance than samples with more Cd, but this trend was appropriate when the molar ratio of In:Cd was 0.6:0.4 (Figure 6a). This may be due to excessive Cd as photocorrosion will occur, resulting in the deterioration of PEC performance.^[^ ^38^ ^]^ To further understand the role of the buffer layer in the photocathode, the following test means will be further understood.

In the field of photocatalytic hydrogen production, one of the core problems that experts have been focusing on is how to improve the separation efficiency of photogenerated carriers.^[^ ^39^ ^]^ However, we know that the reasonable design of the buffer layer can have a useful effect on charge separation. It is particularly important to evaluate the charge separation ability before hydrogen production experiments of photocathode Pt‐TiO_2_/In*_x_* Cd_1−_ *_x_* S/Cu_3_BiS_3_ under sunlight. Photochemical measurements were first performed to elucidate the effective charge separation of the five photocathodes (as shown in Figure 6c). First, electrochemical impedance spectroscopy (EIS) measurements were used to gain an in‐depth understanding of the charge transfer dynamics of different buffer layers in Pt‐TiO_2_/In*_x_* Cd_1−_ *_x_* S/Cu_3_BiS_3_ photocathodes. This test was performed under AM 1.5G illumination at a bias of 0.3 *V*_RHE_. The equivalent circuit model was further used for detailed understanding and consisted of two resistive capacitor blocks in series with resistance R, and phasing elements were used to fit the electrochemical impedance spectroscopy (EIS) results.^[^ ^12^, ^40^ ^]^ The fitting resistance results are shown in Table S2, Supporting Information, in which *R*_s_ is mainly composed of contact sheet resistance and external resistance, *R*_1_ is usually affected by carrier transport in the electrode and *R*_2_ represents the impedance at the electrode/electrolyte interface.^[^ ^12^ ^]^ Numerically, the Pt‐TiO_2_/In*_x_* Cd_1−_ *_x_* S/Cu_3_BiS_3_ photocathode covering buffer layer In_0.6_Cd_0.4_S has low resistance values, which is consistent with the minimum semicircle of Pt‐TiO_2_/In_0.6_Cd_0.4_S/Cu_3_BiS_3_ in the Nyquist diagram, indicating that its interfacial charge transfer resistance is the lowest.^[^ ^12^ ^]^ This may be because In_0.6_Cd_0.4_S is a suitable buffer layer for Cu_3_BiS_3_ and can transfer electrons quickly. On the other hand, In_0.6_Cd_0.4_S may combine well with the interface of Cu_3_BiS_3_ and uniformly cover the Cu_3_BiS_3_, resulting in the overall excellent charge transfer performance.

Furthermore, as we predicted, the lower the interfacial transfer resistance of photocathode Pt‐TiO_2_/In_0.6_Cd_0.4_S/Cu_3_BiS_3_ is, the faster the carrier transfer will be. As shown in Figure SS12 Supporting Information, IMPS suggested that the carrier transfer time of the samples. We found that the carrier transfer time of the Pt‐TiO_2_/In_0.6_Cd_0.4_S/Cu_3_BiS_3_ photocathode was shorter than that of the other four photocathodes, indicating that the insertion of an appropriate buffer layer into the photocathode can effectively improve the charge transfer efficiency.^[^ ^41^ ^]^

Moreover, the transient photocurrent spectrum showed that the photocurrent signal of the Pt‐TiO_2_/In_0.6_Cd_0.4_S/Cu_3_BiS_3_ photocathode exhibited a greatly enhanced photocurrent signal compared to the other photocathodes, especially Pt‐TiO_2_/In_2_S_3_/Cu_3_BiS_3_ (as shown Figure 6d). It was revealed that a buffer layer with an appropriate ratio of In and Cd will promote the generation and transfer of photocathode electron hole pairs. Furthermore, from the perspective of current normalization, the Pt‐TiO_2_/In_0.6_Cd_0.4_S/Cu_3_BiS_3_ photocathode showed relatively less photocurrent loss and a wider saturated photocurrent region at the same time than the other photocathodes, which indicates that carrier recombination was less in the Pt‐TiO_2_/In_0.6_Cd_0.4_S/Cu_3_BiS_3_ photocathode and that the carrier lifetime was also longer.^[^ ^40^ ^]^ This can be further illustrated from IMVS.

IMVS is shown in Figure 6e and Figure S13, Supporting Information. With the increase in LED light intensity, the carrier life of the five photocathodes gradually tends to be stable. This is due to LED with higher light intensity illuminating at the photocathode and the light excitation of carriers and their recombination rate tend to balance leading to the gradual stability of carrier life.^[^ ^12^ ^]^ In particular, the Pt‐TiO_2_/In_0.6_Cd_0.4_S/Cu_3_BiS_3_ photocathode showed a higher carrier lifetime, while the Pt‐TiO_2_/In_2_S_3_/Cu_3_BiS_3_ photocathode showed the lowest carrier lifetime, which again indicates that when Cu_3_BiS_3_ has an appropriate buffer layer, the recombination of carriers at the interface will be reduced and its carrier lifetime will be extended.

In the current electrode systems, it is a challenge to maintain long‐term stability, especially in the case of a high current. In this work, Pt‐TiO_2_/In_0.6_Cd_0.4_S/Cu_3_BiS_3_ and Pt‐TiO_2_/In_2_S_3_/Cu_3_BiS_3_ were covered with TiO_2_ of the same thickness. We surprisingly found from **Figure** **2**a that the Pt‐TiO_2_/In_0.6_Cd_0.4_S/Cu_3_BiS_3_ photocathode not only had a higher photocurrent but also had a better stability than Pt‐TiO_2_/In_2_S_3_/Cu_3_BiS_3_. Specifically, the Pt‐TiO_2_/In_0.6_Cd_0.4_S/Cu_3_BiS_3_ photocathode had a better performance, and we can see that after 6 h of the durability test, the photocathode still had a high photocurrent of 10 mA cm^−2^, and the photocurrent did not decrease significantly (as shown in Figure S15, Supporting Information). However, we observed that even though Pt‐TiO_2_/In_2_S_3_/Cu_3_BiS_3_ had a considerable photocurrent at the initial stage, the current density decreased to 3 mA cm^−2^ after the 6 h durability test. This is mainly because the internal charge separation of the Pt‐TiO_2_/In_2_S_3_/Cu_3_BiS_3_ electrode was relatively slow, and the charge was difficult to transfer out (the relevant proof was as mentioned above), resulting in a large amount of charge accumulation, which corroded the electrode itself.^[^ ^42^ ^]^

To further explore, the surface SEM images of the two samples were characterized before and after the test (Figure S14, Supporting Information). The surface of the Pt‐TiO_2_/In_0.6_Cd_0.4_S/Cu_3_BiS_3_ photocathode was compact before the test (Figure S14a, Supporting Information), and the surface morphology showed little difference after 6 h of testing (Figure S14b, Supporting Information). However, the surface of the Pt‐TiO_2_/In_2_S_3_/Cu_3_BiS_3_ was relatively flat and compact before the test (Figure S14c, Supporting Information). After 6 h of testing, there were obvious cracks (Figure S14d, Supporting Information), indicating that the interface integration of In_2_S_3_ and Cu_3_BiS_3_ may be poor, resulting in corrosion of the photocathode after 6 h of testing, and the surface morphology changed greatly.

On the other hand, the XPS spectra of the TiO_2_/In_0.6_Cd_0.4_S/Cu_3_BiS_3_ sample before and after the durability test are shown in Figure S16, Supporting Information, we did not find significant changes before and after the stability test. It was found that no obvious Bi or Cd peaks were observed before and after the durability test (Figure S16c,d, Supporting Information), indicating that the TiO_2_ film protected the surface of photocathode well during the durability test process. The positions and intensities of the peaks from Ti 2p and O 1s were not significantly changed before and after the durability test (Figure S16a,b, Supporting Information), indicating that the TiO_2_ film has high stability. Besides, the XRD spectra of TiO_2_/In_0.6_Cd_0.4_S/Cu_3_BiS_3_ before and after the durability test were also having no significant changes (Figure S17, Supporting Information). On the whole, the In_0.6_Cd_0.4_S which was an appropriate buffer layer for Cu_3_BiS_3_ showed excellent interface integration with Cu_3_BiS_3_ and the components of the Pt‐TiO_2_/In_0.6_Cd_0.4_S/Cu_3_BiS_3_ were bonded closely, which jointly promoted the photocathode to achieve long‐term stability at high current.

Figure 2b shows the amount of H_2_ and O_2_ that was evolved from the Cu_3_BiS_3_‐based photocathode under steady illumination (AM 1.5 G) at 0 *V*_RHE_ bias. The results showed that the Pt‐TiO_2_/In_0.6_Cd_0.4_S/Cu_3_BiS_3_ photocathode (active area 0.49 cm^2^) generates H_2_ at a rate of 92 µmol h^−1^ in a buffer solution with pH 6.5. This experiment confirmed that the photocathode had great stability. It is worth noting that the Faraday efficiency measured by H_2_ to e^−^/2 was higher than 96%, indicating that the photocurrent was caused by the HER and that no other reduction/oxidation processes occurred on the electrode.^[^ ^43^ ^]^ Therefore, a powerful and highly active water splitting system can be achieved by using the best photocathode and an excellent photoanode. The photograph of the Pt‐TiO_2_/In_0.6_Cd_0.4_S/Cu_3_BiS_3_ photocathode under working, that is, under solar light irradiation at 0 *V*_RHE_ is shown in Figure 2c. We can observe significant H_2_ bubbles produced at the surface of the Pt‐TiO_2_/In_0.6_Cd_0.4_S/Cu_3_BiS_3_ photocathode. Video S1, Supporting Information, is the recorded video of the same sample under solar light irradiation at 0 *V*_RHE_ for solar water splitting reaction.

**Figure 7:**
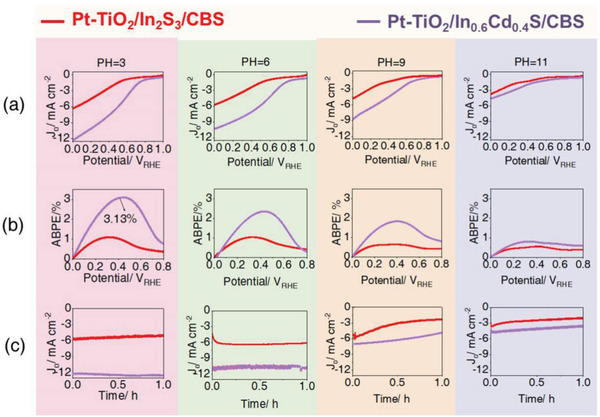
a) Photocurrent density–potential curves; b) corresponding ABPE calculation curves and c) photocurrent densities (0 *V*_RHE_) with illumination time. All figures include typical Pt‐TiO2/In0.6Cd0.4S/Cu3BiS3 (purple) and Pt‐TiO2/In2S3/Cu3BiS3 (red) at different pH values (pH 3.0–11.0) under AM 1.5 G simulated sunlight irradiation.

In our previous report, we found that the photocathode had a different PEC performance in different acidic and alkaline electrolytes.^[^ ^44^ ^]^ Thus far, we have explored the PEC performance of Pt‐TiO_2_/In_0.6_Cd_0.4_S/Cu_3_BiS_3_ and Pt‐TiO_2_/In_2_S_3_/Cu_3_BiS_3_ photocathodes at different pH values (**Figure** **7**). It is observed that Pt‐TiO_2_/In_0.6_Cd_0.4_S/Cu_3_BiS_3_ and Pt‐TiO_2_/In_2_S_3_/Cu_3_BiS_3_ photocathodes both showed an excellent PEC performance in acidic and neutral solution conditions, but with an increase in the pH value, both of these photocathodes showed poor performance, especially at pH 11.0. This is mainly because the formation of H^+^ was accelerated under acidic conditions, and the extraction of excited state electrons by the HER catalyst was promoted.^[^ ^43^ ^]^ Figure S18, Supporting Information, shows that under acidic conditions, the photocurrent loss of the photocathode was the lowest, and there was a wide photocurrent region, which indicates that under acidic conditions, carrier recombination is less.^[^ ^40^ ^]^ However, under alkaline conditions, due to the generation of OH^−^, part of the H^+^ would be neutralized by OH^−^, which would lead to the compound aggravation of carriers. A reduction in the concentration of H^+^ will drive the deterioration of the PEC performance of the photocathode under alkaline conditions. In particular, we found that for the Pt‐TiO_2_/In_0.6_Cd_0.4_S/Cu_3_BiS_3_ photocathode in pH 3.0 buffer solution, the photocurrent density was 12.20 mA cm^−2^ at 0 *V*_RHE_, and the opening potential was 0.9 *V*_RHE_ (Figure 7a). According to the *J*–*V* curve calculation shown in Figure 7b, the ABPE corresponding to Pt‐TiO_2_/In_0.6_Cd_0.4_S/Cu_3_BiS_3_ in pH 3.0 buffer solution exceeded the highest record of 3.13%. This is the highest ABPE value for Cu_3_BiS_3_‐based photocathodes to date. Moreover, we tested the PEC stability of the Pt‐TiO_2_/In_0.6_Cd_0.4_S/Cu_3_BiS_3_ and Pt‐TiO_2_/In_2_S_3_/Cu_3_BiS_3_ photocathodes as a function of the solution pH (as shown in Figure 7c). We observed that the Pt‐TiO_2_/In_0.6_Cd_0.4_S/Cu_3_BiS_3_ and Pt‐TiO_2_/In_2_S_3_/Cu_3_BiS_3_ photocathodes showed more stable PEC performance in acidic (pH 3.0) and neutral (pH 6.5) electrolytes, while the photocurrent of the same sample showed that the current density was low and unstable in alkaline solution (pH 9.0, pH 11.0). Furthermore, it is worth noting that at different pH values, the stability of the Pt‐TiO_2_/In_0.6_Cd_0.4_S/Cu_3_BiS_3_ photocathode was better than that of Pt‐TiO_2_/In_2_S_3_/Cu_3_BiS_3_, which was probably because In_0.6_Cd_0.4_S was a better match for Cu_3_BiS_3_ in terms of band matching and could speed up the transfer of electrons to reduce corrosion by itself.

**Figure 8:**
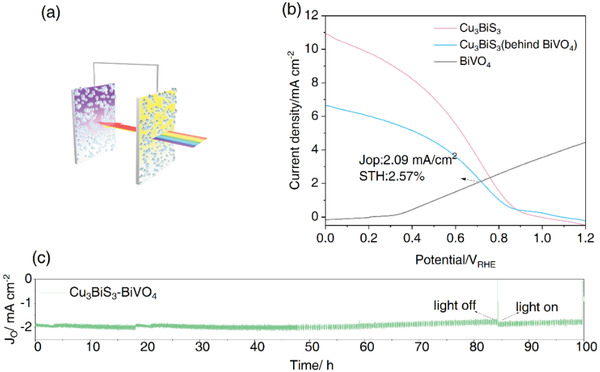
Solar‐driven overall water splitting PEC cell. a) Schematic diagram of the Pt‐TiO_2_/In_0.6_Cd_0.4_S/Cu_3_BiS_3_‐BiVO_4_ tandem cell; b) LSV curves of the Pt‐TiO_2_/In_0.6_Cd_0.4_S/Cu_3_BiS_3_ photocathode and BiVO_4_ photoanode; c) unbiased photocurrent density of the Pt‐TiO_2_/In_0.6_Cd_0.4_S/Cu_3_BiS_3_ ‐BiVO_4_ tandem cell.

It is noteworthy that the Cu_3_BiS_3_‐based photocathode has a high initial potential of 0.9 *V*_RHE_, which is of great significance for the efficient series assembly of photocathode–photoanode cells. In this work, we prepared a Cu_3_BiS_3_‐BiVO_4_ series cell. As shown in **Figure** **8**a, the BiVO_4_/FTO photoanode was placed in front of the Pt/TiO_2_/In_0.6_Cd_0.4_S/Cu_3_BiS_3_ photocathode under AM 1.5 G simulated solar radiation, and then this double‐electrode tandem PEC cell was assembled. Therefore, as Figure 8b shows, the *J*–*V* curves of the Cu_3_BiS_3_ photocathode and BiVO_4_ photoanode overlap obviously, and the intersection point/operation photocurrent density of the tandem cell is 2.09 mA cm^−2^. Using the equation *n*_STH_ = (*J*_op_ × 1.23)/*P* (*P* is the illumination power), we were surprised to find that the STH of this Cu_3_BiS_3_‐BiVO_4_ tandem cell achieved a remarkable efficiency of 2.57%, which is the highest unbiased STH efficiency reported for the Cu_3_BiS_3_‐BiVO_4_ series cell. We can see a lot of H_2_ and O_2_ bubbles produced at the Cu_3_BiS_3_ photocathode and BiVO_4_ photoanode only under solar light soaking without bias. (Video S2, Supporting Information)

Stability is another important criterion for evaluating device performance. It was found that our Pt‐TiO_2_/In_0.6_Cd_0.4_S/Cu_3_BiS_3_‐BiVO_4_ tandem cell presented quite high long‐term stability after 100 h of solar light irradiation (Figure 8c). Compared with typical devices reported previously, the Pt‐TiO_2_/In_0.6_Cd_0.4_S/Cu_3_BiS_3_‐BiVO_4_ tandem cell in our work is very competitive in both STH efficiency and stability (**Figure** **4**), which indicates that the Cu_3_BiS_3_ photocurrent has great application prospects.

## 3 Conclusions

In summary, we researched the influence of interfacial properties between the Cu_3_BiS_3_ film and In*_x_* Cd_1−_ *_x_* S buffer layer for photoelectrochemical water splitting. In this study, we found that the In*_x_* Cd_1−_ *_x_* S buffer layer significantly lowered the CBO of the CBS/buffer and that the rate at introducing In element into CdS of buffer influenced the CBO value of the CBS/buffer. The Pt‐TiO_2_/In_0.6_Cd_0.4_S/Cu_3_BiS_3_ photocathode was found to have an excellent PEC current density of ≈12.20 mA cm^−2^ at 0 *V*_RHE_ and an onset potential of more than 0.9 *V*_RHE_. Another finding is that the ABPE of our Cu_3_BiS_3_‐based photocathode reaches the highest recorded level of 3.13% to date. Furthermore, in terms of stability, this photocathode has a long‐term high stability of over 10 h. By application of the In_0.6_Cd_0.4_S buffer, the Cu_3_BiS_3_‐BiVO_4_ tandem cell presented a stable and excellent unbiased STH of 2.57% for over 100 h. Therefore, we believe that the solar hydrogen conversion efficiency, unbiased water separation efficiency and stability of the Cu_3_BiS_3_‐based photocathode will be improved again through systematic optimization using interface engineering, band orientation, carrier transport and other technologies.^[^ ^45^ ^]^ Further systematic optimization and other studies of this material are underway.

## 4 Experimental Section

Experimental procedures and characterization details are presented in the Supporting Information.

## Conflict of Interest

The authors declare no conflict of interest.

## Supporting information

Supporting Information

Supplemental Video 1

Supplemental Video 2

## Data Availability

The data that support the findings of this study are available from the corresponding author upon reasonable request.
