# Supplementary material for: Research on the Influence of the Interfacial Properties Between a Cu3BiS3 Film and an In x Cd1− x S Buffer Layer for Photoelectrochemical Water Splitting
Source: Adv Sci (Weinh). 2022 Oct 17;9(33):2204029. doi: 10.1002/advs.202204029 (PMC9685470; doi:10.1002/advs.202204029)
Supplement: Supplementary file 1 — Supporting Information [file ADVS-9-2204029-s001.pdf]

ADVANCED SCIENCE
Open Access

Supporting Information

for *Adv. Sci.*, DOI 10.1002/advs.202204029

Research on the Influence of the Interfacial Properties Between a Cu_3BiS_3 Film and an $In_xCd_{1-x}S$ Buffer Layer for Photoelectrochemical Water Splitting

*Xiaomin Wu, Weidong Zhao, Yucheng Hu, Guohong Xiao, Huanyang Ni, Shigeru Ikeda, Yunhau Ng and Feng Jiang**

Supporting Information

Research on the influence of interfacial properties between Cu_3BiS_3 film and $In_xCd_{1-x}S$ buffer layer for photoelectrochemical water splitting

Xiaomin Wu[a], *Weidong Zhao*[a], *Yucheng Hu*[a], *Guohong Xiao*[a], *Huanyang Ni*[a] *and, Shigeru Ikeda*[d], *Yunhau Ng*[e], *Feng Jiang*[a b c]*

a. Institute of Hydrogen Energy for Carbon Peaking and Carbon Neutralization, School of Semiconductor Science and Technology, South China Normal University, Foshan, 528225, China.
b. Institute of Semiconductor Science and Technology, South China Normal University, 55 Zhongshan Avenue West, Tianhe District, Guangzhou 510631, China
c. Key Laboratory of Polar Materials and Devices, Ministry of Education, East China Normal University, Information Building, 500 Dongchuan Road, Shanghai 200241.
d. School of Energy and Environment, City University of Hong Kong, Kowloon, Hong Kong 999077, China
e. Department of Chemistry, Konan University, 9-1 Okamoto, Higashinada, Kobe, Hyogo 658-8501, Japan

*Corresponding Author, Email address: fengjiangsolar@126.com (F. Jiang)

Experimental Details:

Preparation of the Cu_3BiS_3 films.

The solution is made up of $BiCl_3$-Tu DMSO solution (15 mL) which was consists of $BiCl_3$(3.15 g) and an overdose thiourea (Tu) and CuCl-Tu DMSO solution (15 mL) which was formed by CuCl (2.97 g)and an overdose thiourea (Tu). During the operation which followed, the two bottles of solution were stirred separately for a period of time until they are mix well separately. Then mix $BiCl_3$-Tu DMSO solution to CuCl-Tu DMSO and stired for 3 hours. Finally, the precursor

solution was sprayed on clean Mo-coated sodalime glass at 390°C for 4-5 minutes.

Surface modification with $ln_xCd_{1-x}S$ buffer layer.

First, a $ln_xCd_{1-x}S$ layer was deposited under the Cu_3BiS_3 layer by the chemical bath deposition technique (CBD) for a certain amount of time. Specially, the solution of $ln_xCd_{1-x}S$ included $In_2S_3O_{12}$ (99.99%) (0∽12.5 mM), $CdSO_4$ (99.99%) (12.5 mM), $SC(NH_2)_2$(99.99%) (0.3 M) and CH_3COOH (99.99%) (11 M) at 71°C. Next, the TiO_2 layer was deposited under the $ln_xCd_{1-x}S$ /Cu_3BiS_3 double layer by atomic layer deposition (ALD) technique. Notably, TiO_2 film with thickness of 50 nm was vapor deposition using titanium tetroxide (dimethylamine) as titanium source and H_2O as oxygen source. Finally, a TiO_2/ $ln_xCd_{1-x}S$ /Cu_3BiS_3 electrode was obtained.

Deposition of Pt

We used three electrodes for deposition in Pt solution which was prepared by the Na_2SO_4 solution (0.1 M) and H_2PtCl_6 (1 Mm), among which, Ag/AgCl was the reference electrode, Pt was the opposite electrode and TiO_2/ $ln_xCd_{1-x}S$ /Cu_3BiS_3 was the working electrode. In particular, the electrode system deposited 10s under the irradiation of AM 1.5G.

Structural characterization

The crystalline structure of the Cu_3BiS_3 film was confirmed by X-ray diffraction (XRD) using a Rigaku Mini Flex X-ray diffractometer. Surface and cross section morphology was exhibited by scanning electron microscope (SEM) using Hitachi S-4800 microscope. UV–vis absorption spectra were illustrated by a UV–Vis NIR spectrophotometer. X-ray photoelectron spectroscopy (XPS) using a Shimadzu AXIS ULTRA X-ray photo-electron spectrometer. TEM using a JEOL JEM2100HR microscope.

Photoelectrochemical measurements

The data of photoelectrochemical properties were detected by the following

equipment. First, the CHI660E electrochemical workstation was used to test LSV in a buffer solution (pH = 6.5) under AM 1.5 G sunlight irradiation. Moreover, we test impedance and then the fitting is carried out on Zview. Finally, an online gas chromatography system was used to detect H_2 and O_2 in water splitting under simulated light AM 1.5G.

In the experiment, the potential is converted by the Ag/AgCl electrode potential into reversible hydrogen electrode by Nernst equation

$$V_{RHE}=V_{Ag/AgCl}+0.059\times PH+0.199$$

ABPE was determined by the current-density-potential response of the photocathode, and the formula is as follows:

$$ABPE(\%)=J\times V\times 100/P$$

Where J is the photocurrent density (mA/cm^2), V is the applied potential (V_{RHE}), and P is the intensity of simulated sunlight (100 mW/cm^2).

In the IMPS test，the charge transfer time τ_d can be obtain from using the following equation:

$$\tau_d = 1/(2\pi f_{min})$$

Where the f_{min} is the lowest point in the IMPS plots

The photoexcited carrier lifetimes of photocathode can be calculated from the IMVS plots as the followed equation:

$$\tau_n = 1/(2\pi f_{min})$$

Where the f_{min} is the lowest point in the IMVS plots.

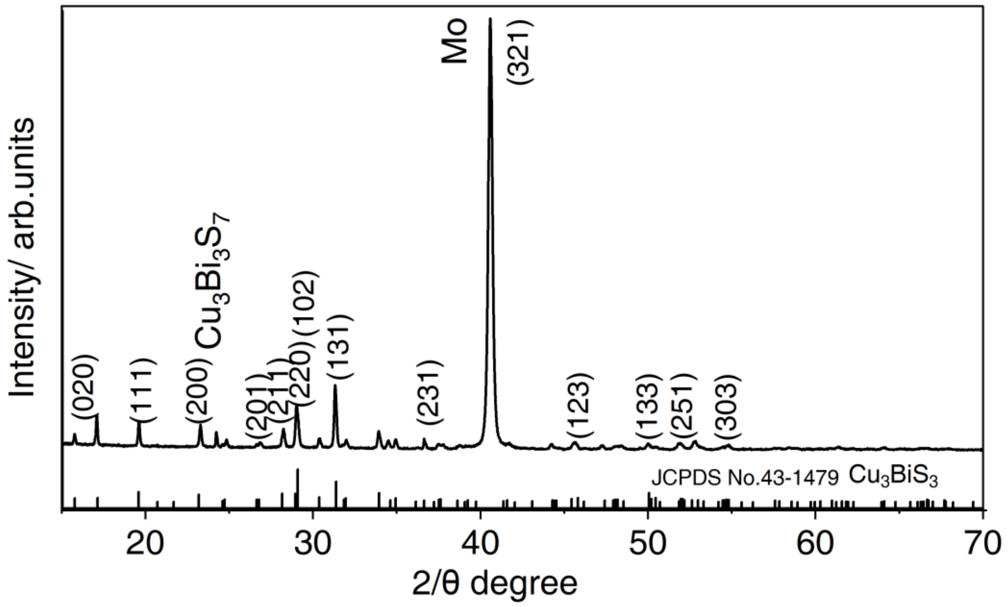

Figure S1 XRD pattern of the Cu_3BiS_3 films.

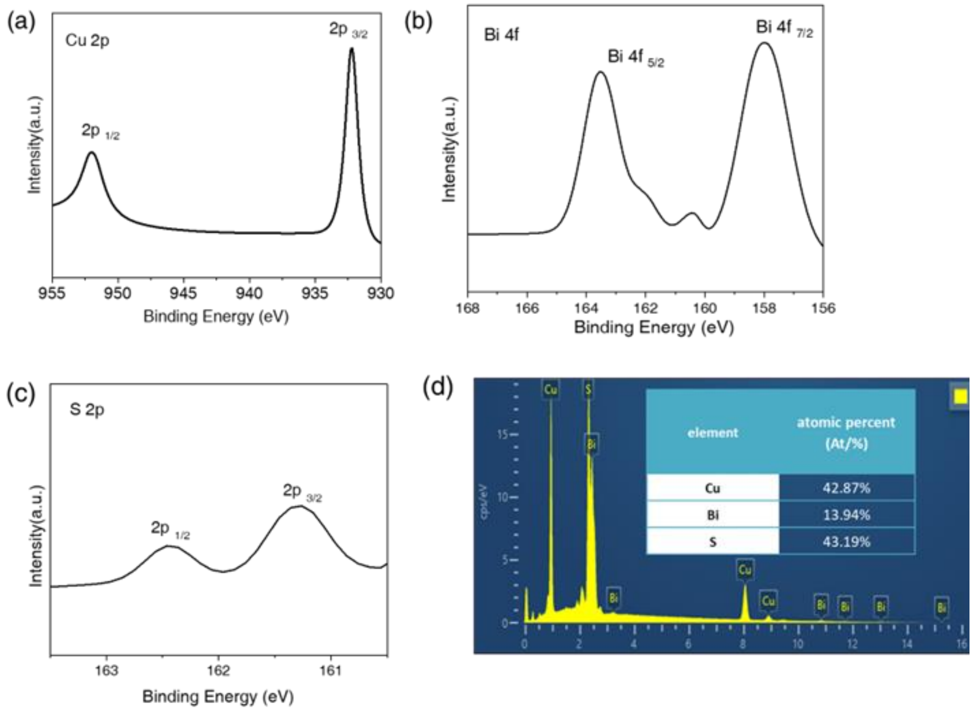

Figure S2 XPS of the Cu_3BiS_3 (a) Cu 2p;(b) Bi 4f; (c) S 2p; (d) EDS of Cu_3BiS_3

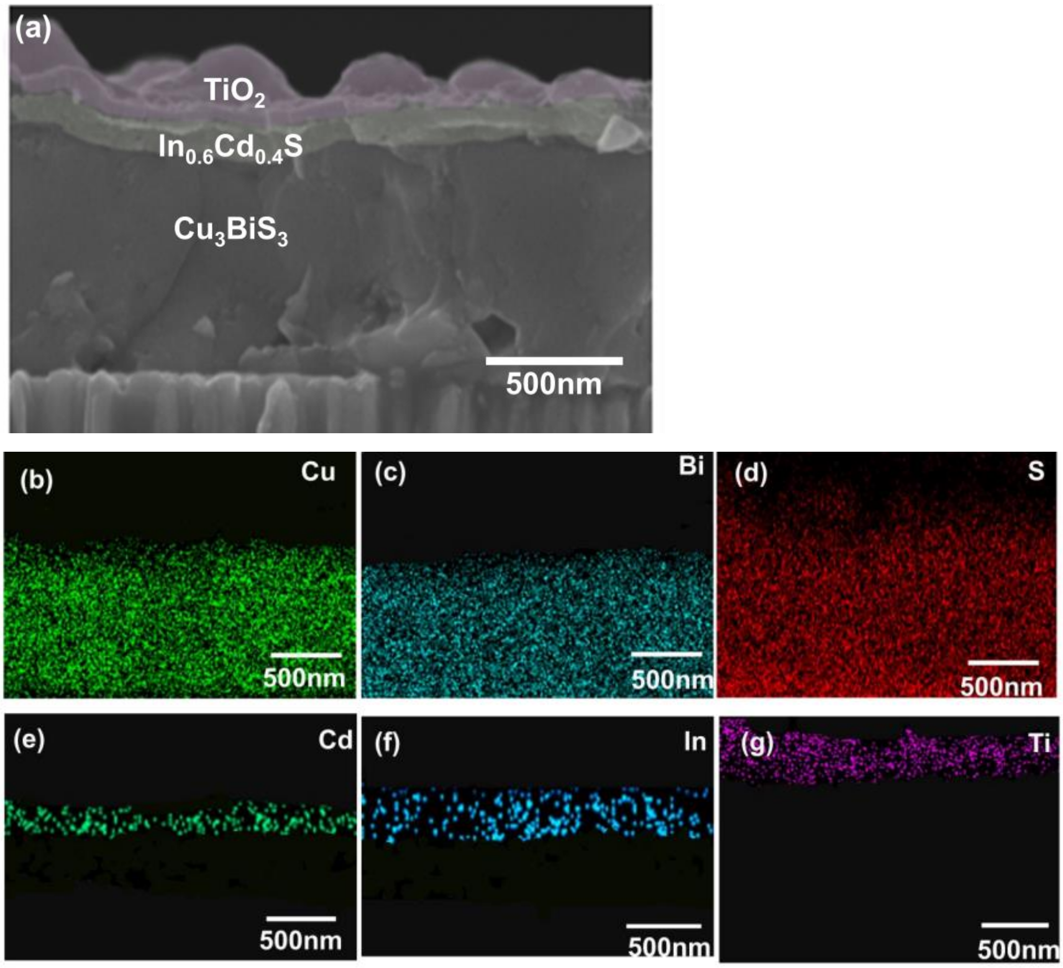

Figure S3 (a) Cross-section SEM images of the Pt-TiO_2/$In_{0.6}Cd_{0.4}S$/Cu_3BiS_3 electrode; EDS mapping for the elements of (b) Cu, (c) Bi, (d) S, (e) Cd, (f) In, (g) Ti in the whole area of (a)

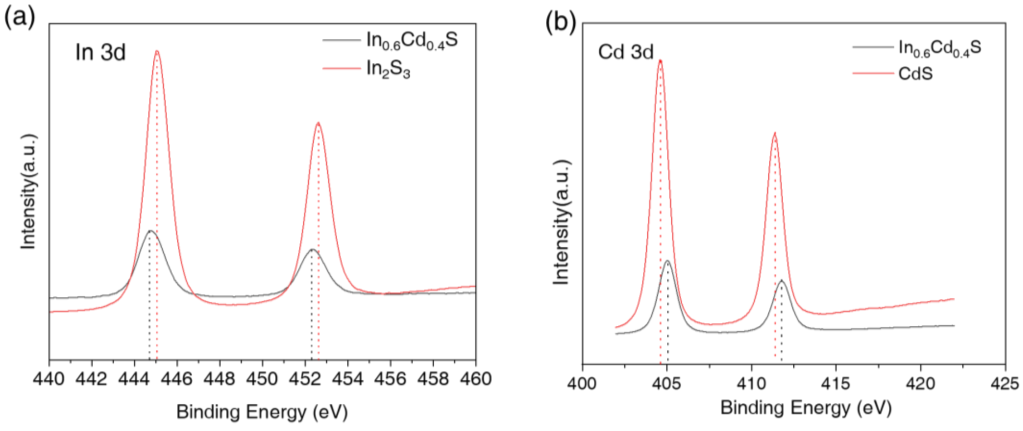

Figure S4 XPS (a) In 3d of $In_{0.6}Cd_{0.4}S$ and In_2S_3 ;(b) Cd 3d of $In_{0.6}Cd_{0.4}S$ and CdS

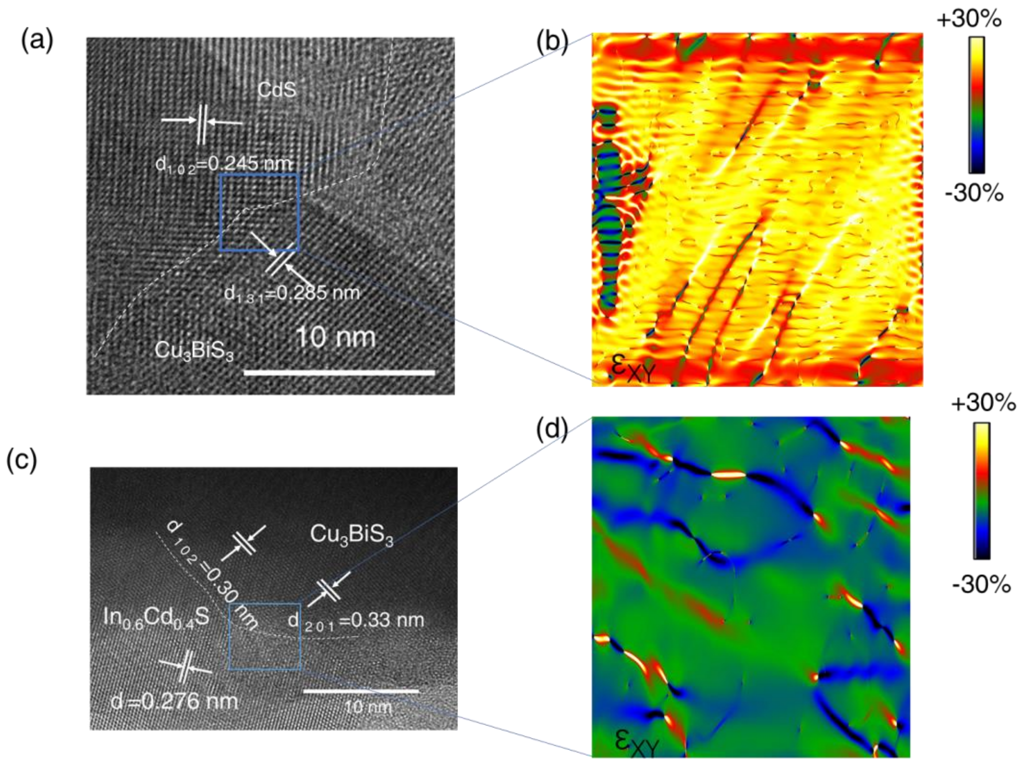

Figure S5 (a) high-resolution HRTEM image of Cu_3BiS_3/CdS; (b) strain mapping of Cu_3BiS_3/CdS heterostructure (ε_{xy} denotes the strain along the xy direction). (c) high-resolution HAADF-STEM image of Cu_3BiS_3/$In_{0.6}Cd_{0.4}S$; (d) strain mapping of Cu_3BiS_3/$In_{0.6}Cd_{0.4}S$ heterostructure (ε_{xy} denotes the strain along the xy direction).

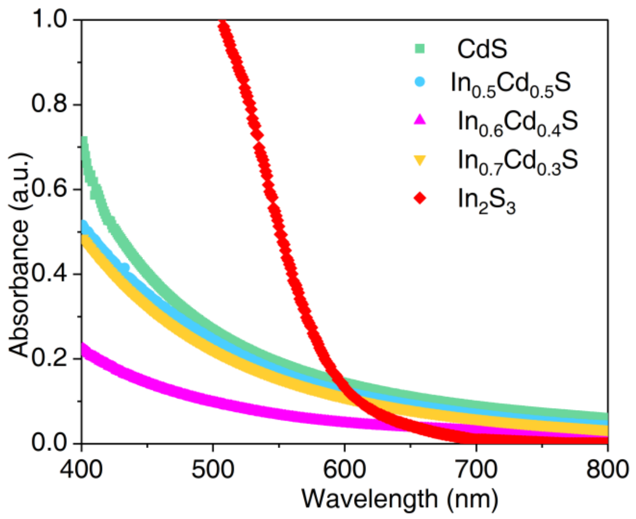

Figure S6 The absorption spectra of CdS, $In_{0.5}Cd_{0.5}S$, $In_{0.6}Cd_{0.4}S$, $In_{0.7}Cd_{0.3}S$, In_2S_3

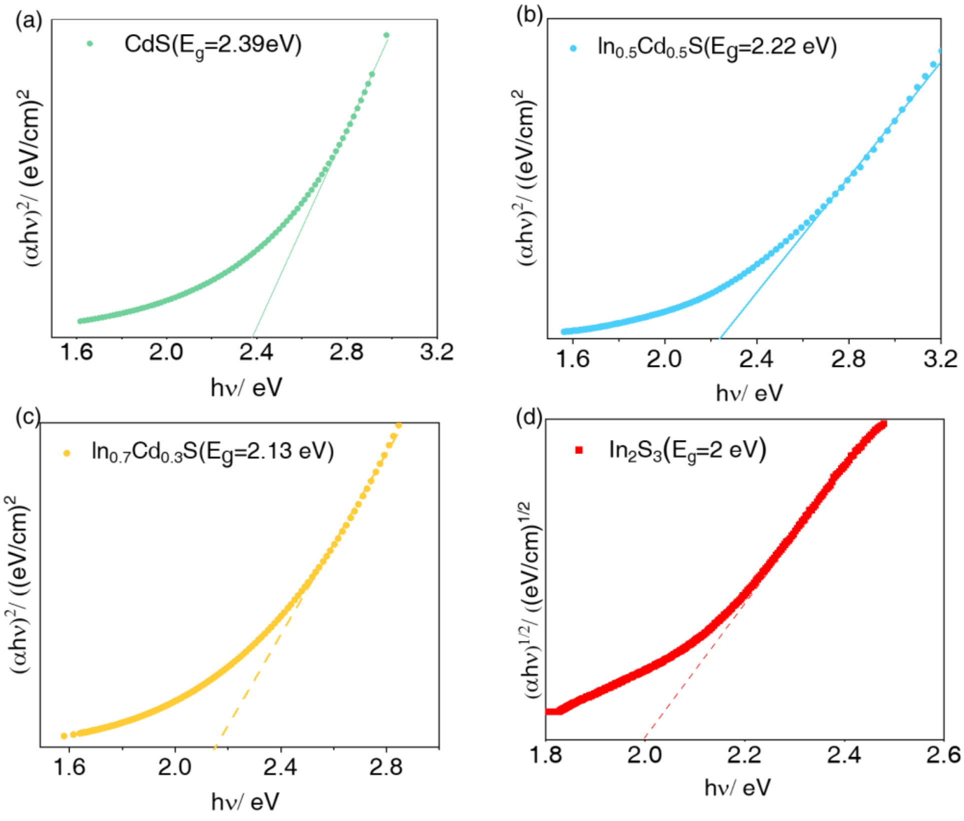

Figure S7 Tauc curve of (a) CdS; (b) $In_{0.5}Cd_{0.5}S$; (c) $In_{0.7}Cd_{0.3}S$; (d) In_2S_3.

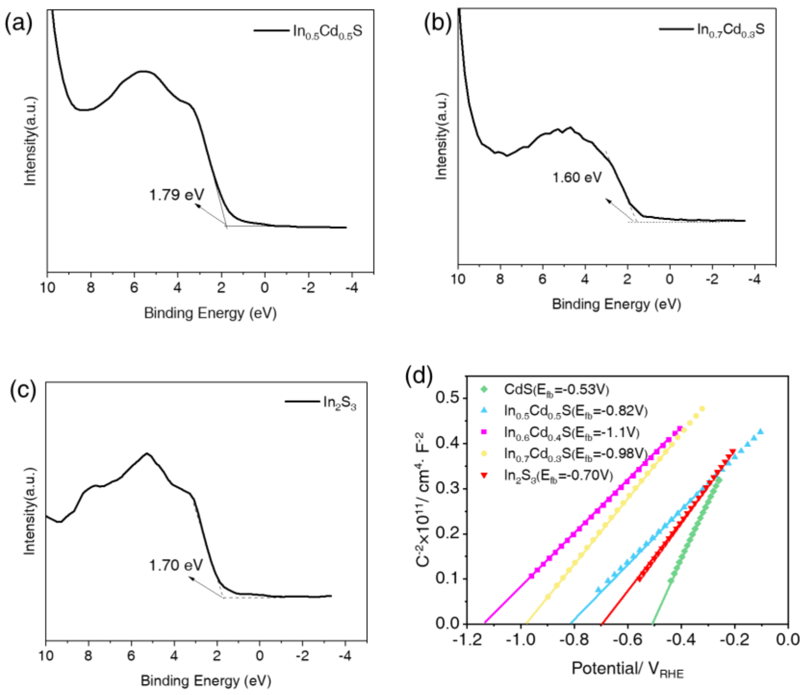

Figure S8 (a) VB-XPS of $In_{0.5}Cd_{0.5}S$; (b) VB-XPS of $In_{0.7}Cd_{0.3}S$; (c) VB-XPS of In_2S_3; (d) Mott-Schottky plot of CdS, $In_{0.5}Cd_{0.5}S$, $In_{0.6}Cd_{0.4}S$, $In_{0.7}Cd_{0.3}S$, and In_2S_3 in the dark.

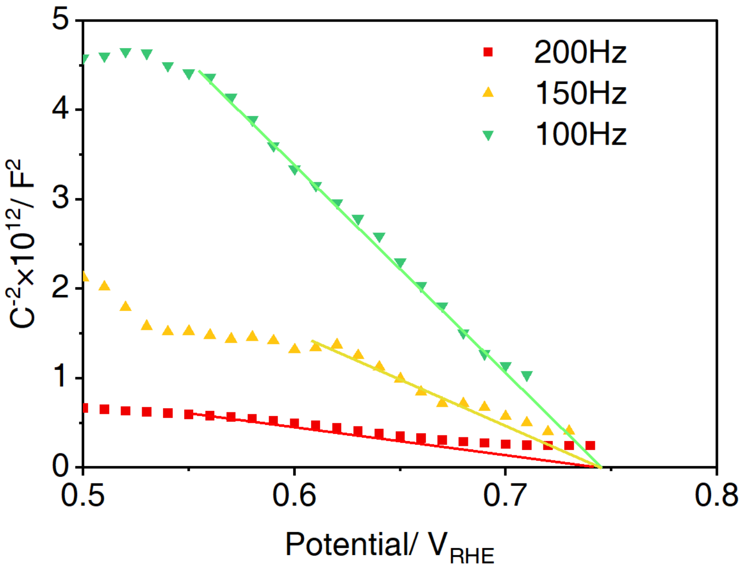

Figure S9 Mott-Schottky plot of Cu_3BiS_3 in different frequencies in the dark.

Table S1. Band structure of the different samples

Sample	$E_{VB, XPS}$	$E_{VB, NHE}$	E_g	$E_{CB, NHE}$= ($E_{VB, NHE}$) -E_g
Cu_3BiS_3	0.88	0.64	1.67	-1.03
CdS	2.30	2.06	2.39	-0.33
$In_{0.5}Cd_{0.5}S$	1.79	1.55	2.22	-0.67
$In_{0.6}Cd_{0.4}S$	1.54	1.30	2.18	-0.88
$In_{0.7}Cd_{0.3}S$	1.60	1.36	2.13	-0.77
In_2S_3	1.70	1.46	2.00	-0.54

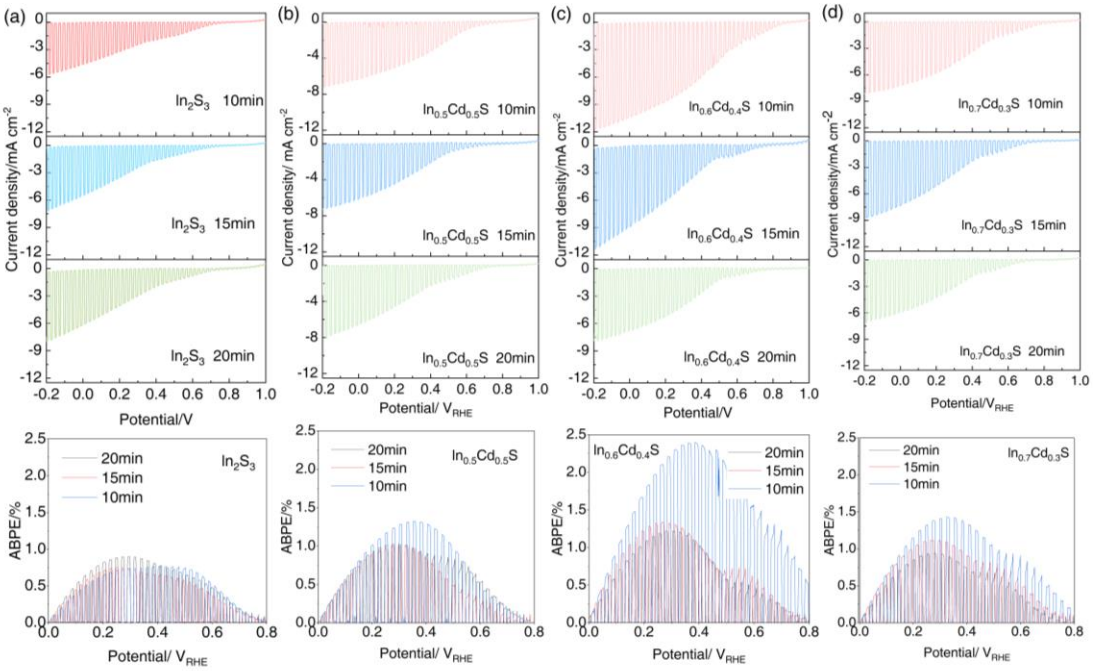

Figure S10 The PEC performance of Cu_3BiS_3 based photocathode with different thicknesses of In_2S_3, $In_{0.5}Cd_{0.5}S$, $In_{0.6}Cd_{0.4}S$, $In_{0.7}Cd_{0.3}S$ and their corresponding ABPE. (a) is the Pt-TiO_2/In_2S_3(differ thickness)/Cu_3BiS_3 photocathode; (b) is the Pt-TiO_2/$In_{0.5}Cd_{0.5}S$/Cu_3BiS_3 photocathode; (c) is the Pt-TiO_2/$In_{0.6}Cd_{0.4}S$/Cu_3BiS_3 photocathode; (d) is the Pt-TiO_2/$In_{0.7}Cd_{0.3}S$/Cu_3BiS_3 photocathode.

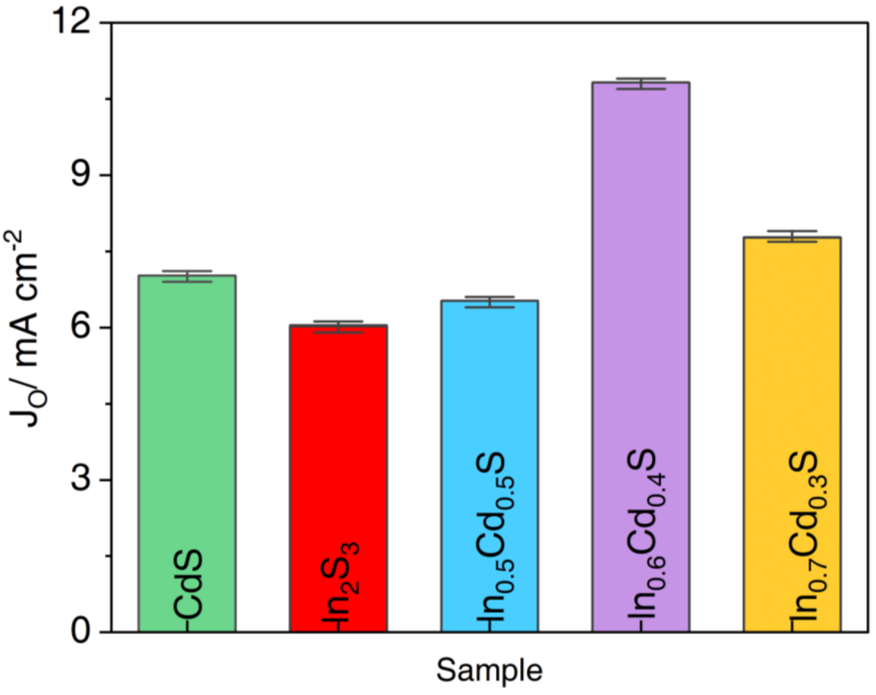

Figure S11 Statistical box data of PEC performance of photocurrent density for the Pt-TiO_2/CdS/Cu_3BiS_3; Pt-TiO_2/In_2S_3/Cu_3BiS_3; Pt-TiO_2/$In_{0.5}Cd_{0.5}S$/Cu_3BiS_3; Pt-TiO_2/$In_{0.6}Cd_{0.4}S$/Cu_3BiS_3; Pt-TiO_2/$In_{0.7}Cd_{0.3}S$/Cu_3BiS_3 photocathodes. The number of the statistical samples of each parameter is 10.

Table S2. Fitted EIS resistance parameters based on the circuits show in the Figure 4c

Sample	R_s/Ω	R_1/Ω	R_2/Ω
$Pt\text{-}TiO_2/CdS/Cu_3BiS_3$	14.30	82.20	33.42
$Pt\text{-}TiO_2/In_{0.5}Cd_{0.5}S/Cu_3BiS_3$	14.12	52.90	95.10
**$Pt\text{-}TiO_2/In_{0.6}Cd_{0.4}S/Cu_3BiS_3$**	**14.01**	**40.54**	**19.72**
$Pt\text{-}TiO_2/In_{0.7}Cd_{0.3}S/Cu_3BiS_3$	14.08	45.20	93.33
$Pt\text{-}TiO_2/In_2S_3/Cu_3BiS_3$	14.11	52.02	437.80

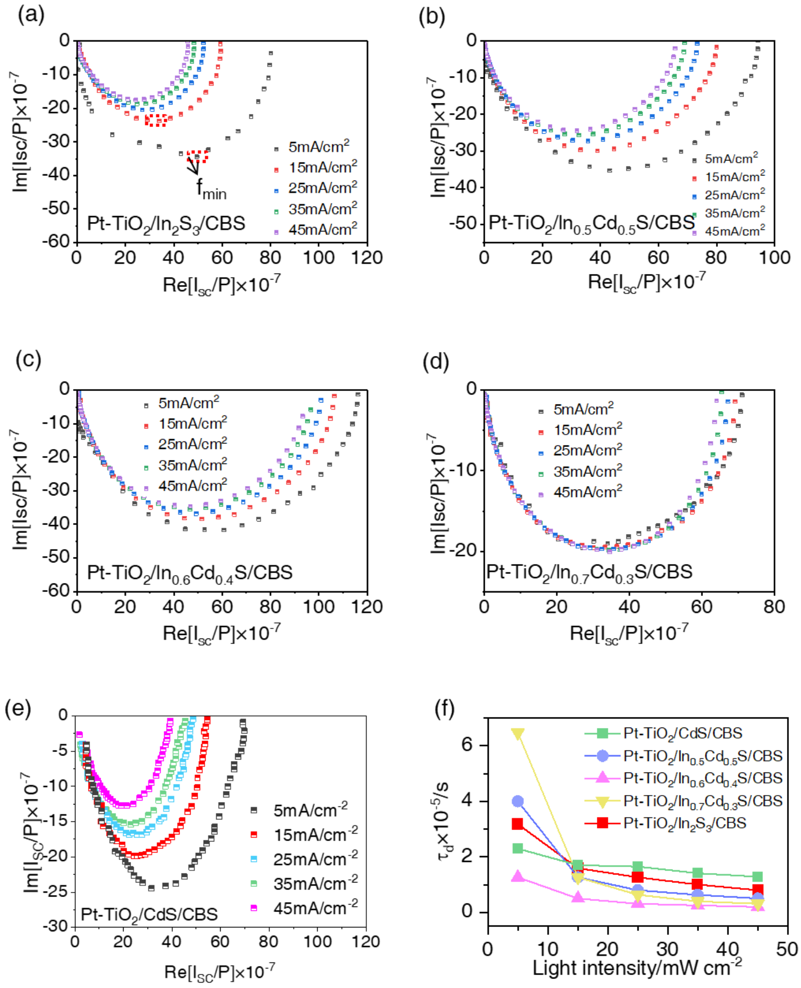

Figure S12 IMPS spectra of (a) $Pt\text{-}TiO_2/In_2S_3/Cu_3BiS_3$; (b)$Pt\text{-}TiO_2/$ $In_{0.5}Cd_{0.5}S/Cu_3BiS_3$; (c) $Pt\text{-}TiO_2/In_{0.6}Cd_{0.4}S/Cu_3BiS_3$; (d) $Pt\text{-}TiO_2/In_{0.7}Cd_{0.3}S/Cu_3BiS_3$; (e) $Pt\text{-}TiO_2/CdS$ $/Cu_3BiS_3$; (f) The calculated transfer time of photoexcited carriers of the sample.

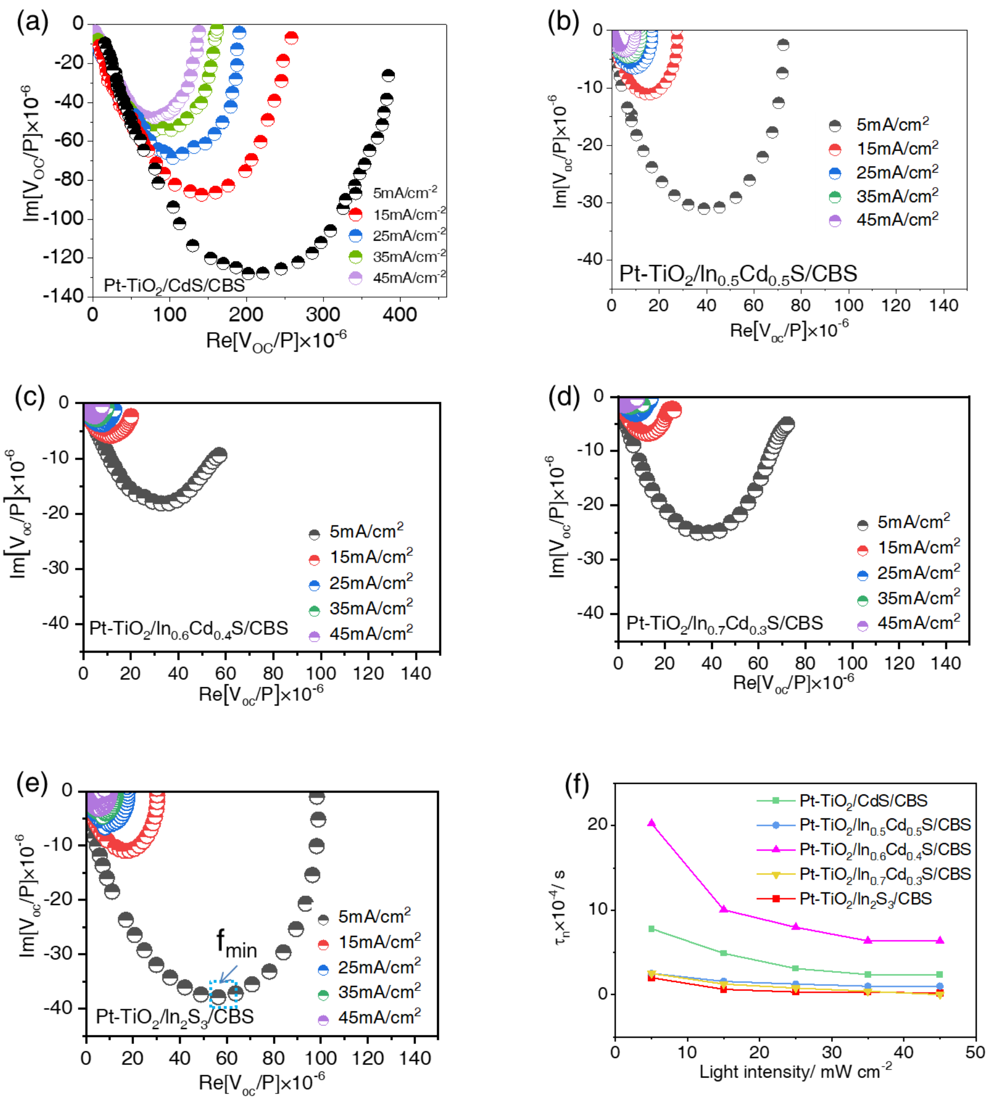

Figure S13 IMVS spectra of (a) Pt-TiO_2/CdS /Cu_3BiS_3; (b)Pt-TiO_2/ $In_{0.5}Cd_{0.5}S$/Cu_3BiS_3; (c) Pt-TiO_2/$In_{0.6}Cd_{0.4}S$/Cu_3BiS_3; (d) Pt-TiO_2/$In_{0.7}Cd_{0.3}S$/Cu_3BiS_3; (e) Pt-TiO_2/In_2S_3/Cu_3BiS_3; (f) the lifetime of carriers generated by these five photocathodes.

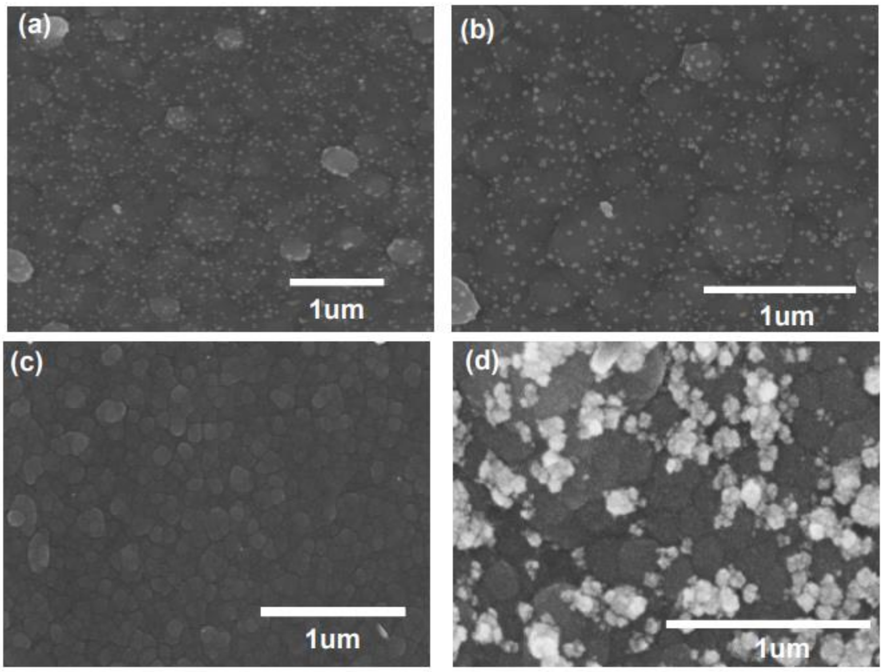

Figure S14 Surface SEM images of Pt/TiO_2/$In_{0.6}Cd_{0.4}S$ /Cu_3BiS_3 (a, b) and Pt/ TiO_2/ In_2S_3/ Cu_3BiS_3 (c, d) photocathodes before (a, c) and after (b, d) a 6 hours durability test.

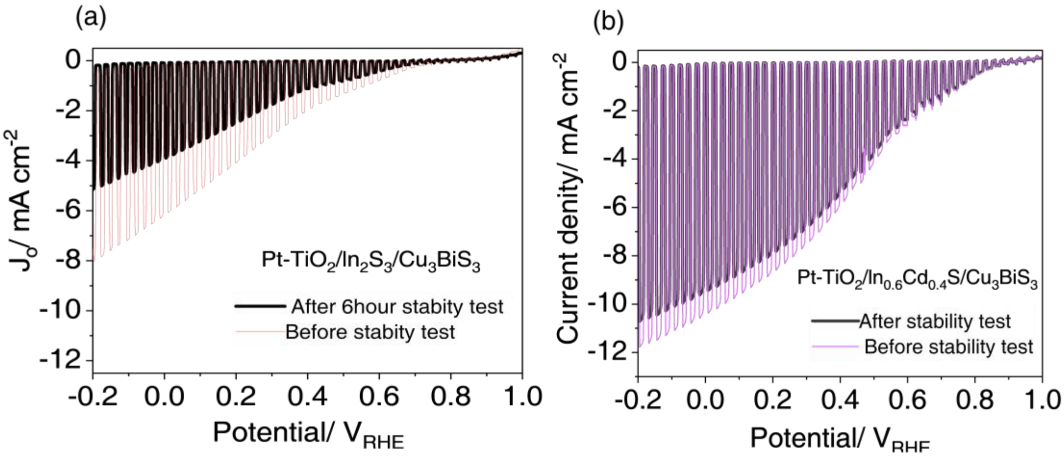

Figure S15 (a) LSV of Pt/TiO_2/In_2S_3/Cu_3BiS_3 and (b) Pt/ TiO_2/$In_{0.6}Cd_{0.4}S$/ Cu_3BiS_3 photocathodes before and after 6 hours durability test.

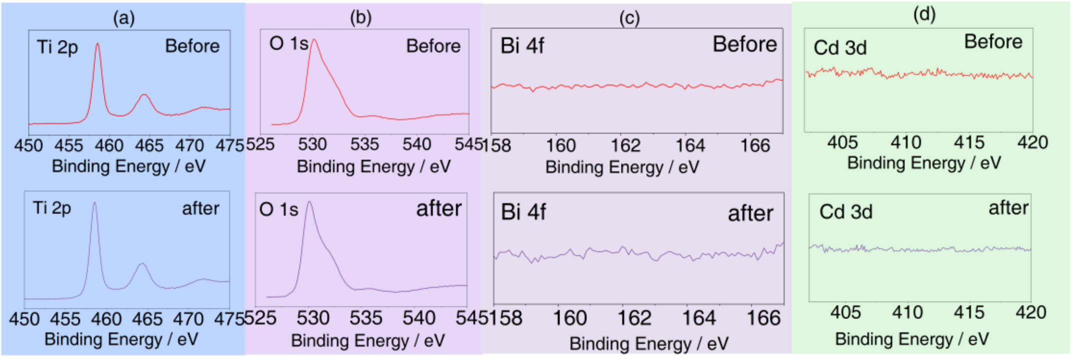

Figure S16 Typical XP spectra from Ti 2p (a), O1s (b), Bi 4f (c) and Cd 3d (d) of Pt/ TiO_2/$In_{0.6}Cd_{0.4}S$/ Cu_3BiS_3 photocathode before and after 6 hours durability test.

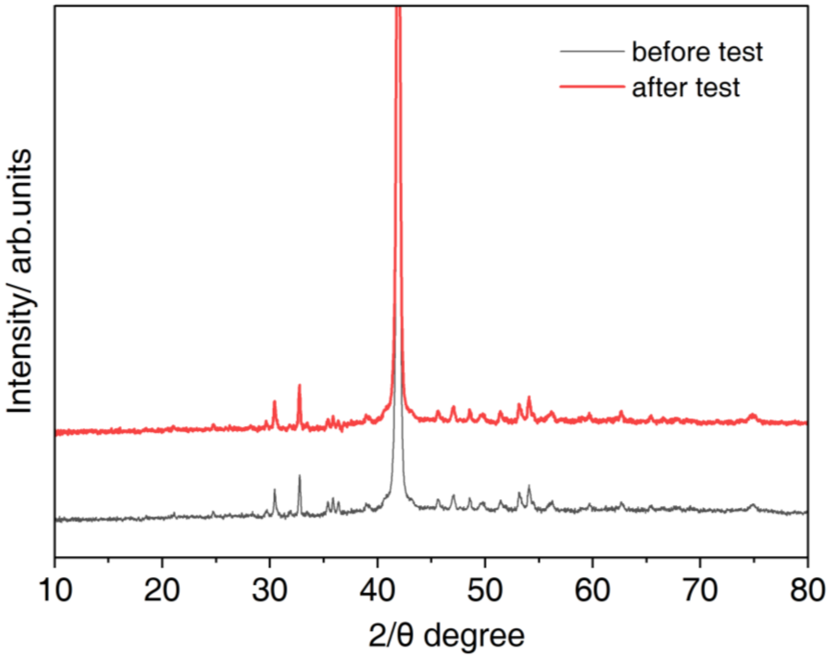

Figure S17 XRD of Pt/ TiO_2/$In_{0.6}Cd_{0.4}S$/ Cu_3BiS_3 photocathodes before and after 6 hours durability test.

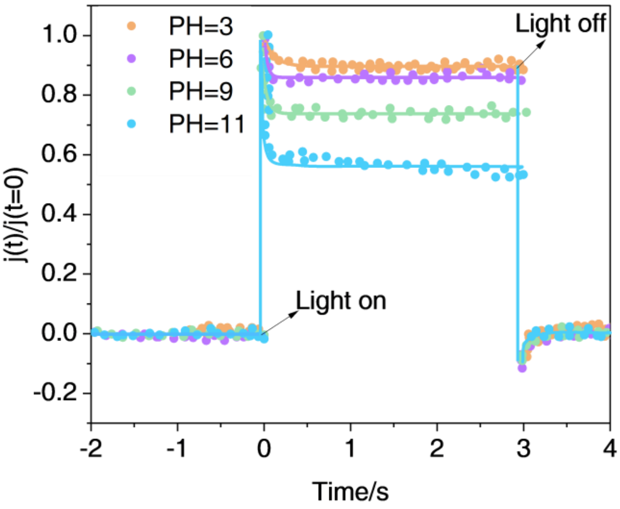

Figure S18 The transit photocurrent spectra of Pt-TiO_2/$In_{0.6}Cd_{0.4}S$/Cu_3BiS_3 in different pH valued (pH 3.0–pH 11.0)
